# Supplementary material for: CODE-EHR best practice framework for the use of structured electronic healthcare records in clinical research
Source: BMJ. 2022 Aug 29;378:e069048. doi: 10.1136/bmj-2021-069048 (PMC9403753; doi:10.1136/bmj-2021-069048)
Supplement: Supplementary file 1 — Web appendix 1: Addressing the need for common standards [file kotd069048.ww1.pdf]

CODE-EHR best practice framework for the use of structured electronic healthcare records in clinical research

Appendix 1: Addressing the need for common standards

The current landscape of digital health records **2**
Electronic healthcare records 2
Coding systems 2
Linkage of coded datasets 3
Common data models 3
Approaches advocated to improve transparency 4

Use of coded healthcare data **4**
Disease definitions 4
Outcome definitions 4

New perspectives for enhanced use of EHR data **5**

Current approaches for assimilating EHR-based evidence **6**
Publication process 6
Regulatory and health technology assessment requirements 7
Implementation to clinical practice 7

Patient concerns and privacy **8**
Social License 8
Public mandate 8
Informed consent 9
Patient and Public involvement 9

References **10**

The current landscape of digital health records

Electronic healthcare records

Electronic healthcare record (EHR) systems were originally designed for business intelligence rather than collection of phenotypes, and can be incomplete with respect to comorbidities not directly related to the medical problem being managed. Heterogeneity exists between different EHR systems in collecting phenotype data. Even within similar platforms, large variations are present due to local and national differences in coding practices. Variability and inaccuracy are introduced when patient data are entered into the EHR and then subsequently coded, demonstrating the need for quality assessment [1]. A major bottleneck is that most of the clinical information is only available as unstructured free text. Natural language processing (text mining) can assist with automatic coding of diseases, but additional research is needed to validate algorithms across different languages, countries and healthcare systems [2].Coordinated efforts are clearly needed from the clinical and patient community in collaboration with industry to discuss and agree on minimal datasets, outcomes measures and standards across different disease domains [3]. Despite these considerable challenges, the exponential rise in the use of EHR systems across the globe provides a clear opportunity to increase the availability of routine data for research purposes.

Coding systems

The procedures for recording patient data in the EHR (and inherent biases) vary from country to country, within countries and according to the coding systems employed. When coded data are used in research, there is often little information available on the coding process, substantially impairing the interpretability of the dataset. Analysis of primary care EHR studies revealed that only 19 of 374 (5.1%) were accompanied by a full set of published clinical codes [4].

Coding systems have been designed for different purposes and employ a variety of versions. The International Classification of Diseases (ICD) catalogue captures diagnoses and is familiar to many clinicians in the context of medical billing. There are numerous derivations of ICD coding, with the most common including ICD-9 (13,000 codes) and ICD-10 (68,000 codes), with a number of countries having their own modifications (note that ICD-11 was launched in 2022 and is designed to avoid for the need for local adaptations). There is considerable variation in how procedures are coded; in the United States the Healthcare Common Procedure Coding System (HCPCS) and ICD-10 Procedure Coding System, in the UK the Office of Population Censuses and Surveys (OPCS) Classification of Interventions and Procedures, and in Europe different versions of the International Classification of Procedures in Medicine (ICPM). In contrast to these classification systems designed to report on outcomes, the Systematised Nomenclature of Medicine Clinical Terms (SNOMED CT) is a controlled clinical terminology that is designed as a structured medical vocabulary for EHRs, with over 300,000 clinical concepts.

The reason and process for coding is critical to understand. Coding may be conducted for clinical service review and/or medical billing purposes and is typically performed by personnel without medical training. In some institutions, clinical oversight and/or clinical audit is part of the coding process in order to improve accuracy [5]. The justification for assigning codes to primary or secondary diagnoses can be ambiguous, and the distinction between pre-existing and new diagnoses may be unclear (i.e. difficulty in differentiating prevalent and incident disease) [6]. Incomplete, non-specific, or imprecise documentation by physicians are major barriers to achieving good data quality and there are numerous nodes in the coding chain that are subject to bias. For example, in an interview study of medical chart coders in Canada, variability was noted in the interpretation of

often incomplete documentation, with the diverse workforce expected to complete a minimum daily quota of charts [7]. Conflicts of interest may emerge when coding is conducted for reimbursement purposes [8]. On the other hand, a study in the UK public health system found that the accuracy of diagnostic coding improved after introducing reimbursement based on coded healthcare data [9]. Countries also have different cultural practices within divergent healthcare models, with varying disease burden and different approaches to how healthcare outcomes are reported.

In summary, coded healthcare data have inherent limitations, including: (1) Missing data and variable quality across and within nations; (2) Quality that depends on who performed the coding and the purpose of coding; (3) Financial aspects and reimbursement that can impact reliability; (4) A lack of consensus on coding systems or codes used for diagnoses and procedures; (5) Imprecision resulting from the lack of standardised variables and coding; and (6) Other sources of bias, including lack of representativeness, misclassification and loss to follow-up.

Linkage of coded datasets

Clinical research often requires data on clinical context (participant demography, characteristics, medical history), healthcare process (therapies, procedures) and outcomes (death, hospitalisation, cause-specific events). Researchers often need to use multiple datasets to generate outcomes for a study population, which may necessitate combining data recorded using different coding systems. In a few countries and regions, citizens have a unique personal identity number which can be used to deterministically link multiple data sources [10]; however in most cases, data linkage is required using non-unique fields that introduces the potential for bias and error. Linkage errors arise when data are missing or inaccurate (false negatives), or unrelated records are mistakenly linked (false positives). Incomplete data on patient and population factors can lead to the potential for systematic bias in reported clinical outcomes [11]. Data pre-processing is a vital aspect of linkage, since many administrative datasets contain inconsistent or incomplete data that vary in structure, format and content [12].

Common data models

Approaches that harmonize data to a common structure, such as common data models, can enable better-quality linkage of multiple data sources and provide a common platform in which analytical methods can be build and deployed across multiple sources. An example is the Observational Health Data Sciences and Informatics (OHDSI) Observational Medical Outcomes Partnership (OMOP) Common Data Model [13] The OMOP Common Data Model consists of 23 tables which are organized in four top-level domains: clinical, derived elements, health system, and health economics. Clinical data tables hold core data on patient demographics, clinical events (e.g., diagnoses, laboratory measurements, medication prescriptions, and surgical procedures), visit occurrences and observation periods. The health system data tables provide information on healthcare providers associated with the healthcare events held in the clinical data types. The health economics data tables contain cost information and details on enrolment of patients in health benefit plans. Individual datasets are transformed into the OMOP Common Data Model through an Extract, Transform & Load (ETL) process which maps data fields in each data source to OMOP vocabulary concepts (Athena, https://athena.ohdsi.org/) and to the Common Data Model schema. Once data are transformed, federated studies can be executed on every source containing data in the Common Data Model, such as the recent COVID-19 studies performed by OHDSI on multiple data partners across the world (for more information, see https://www.ohdsi.org/covid-19-updates/).

Approaches advocated to improve transparency

The FAIR data principles advocate findability, accessibility, interoperability, and reusability as the foundation of good scientific data management and stewardship [14]. Transparency is essential to allow independent scientists to scrutinise data. One of the key challenges lies in the lack of a common approach for defining and validating algorithms that characterize disease status or severity, as well as lifestyle or other risk factors in structured EHRs [15]. This problem could be addressed by internationally agreed standards encapsulating information on phenotypes in a machine-readable manner (including metadata, implementation, validation and terminologies). Computable representations of phenotypes can enable the portability of algorithms across similar sources nationally and internationally, as well as enhance reproducibility by external researchers using the same coded data [16]. Common data models can enable harmonization of information across different EHR sources into a common data schema, however conversion to these models can be challenging and likely requires careful validation to assess potential loss of information fidelity.

Use of coded healthcare data

Coded healthcare data from EHR systems are mainly used in clinical research to define a population of interest with a specific disease or group of conditions, and/or to define a clinical outcome or set of outcomes.

Disease definitions

Studies often define diseases using bespoke code lists, and the processes by which they are developed and validated is not always clear. In research studies, these code lists often define the patient population under investigation; hence it is vital that the processes underpinning patient selection are clear. Funding bodies, journals and regulators have not required researchers to publish their code lists prior to analysis, making it impossible for others to evaluate the validity of the research, replicate the study, or effectively compare studies [4].

At present, the gap in disease definitions is filled by interested research groups, such as the HDR-UK CALIBER Phenotype Library and the Vanderbilt Phenotype Knowledgebase which contain algorithms enabling researchers to reuse disease definitions [17, 18]. The OHDSI community was able to rapidly reuse its definitions to conduct a study on hypertension treatment and COVID-19 [19]. This process has recently been published as a set of 10 principles, called the Large-scale Evidence Generation and Evaluation across a Network of Databases (LEGEND) [20].

There is no consensus on the gold standard for assessing the validity of disease definitions. Although samples of the research population could be validated against case notes, this process is time-consuming, unsuitable for large or diverse samples, and is subject to ascertainment and performance biases. Computer-assisted text mining has clear potential, for example the use of Natural Language Processing to extract and label EHR data, thereby providing substantial granularity that could validate the coded healthcare record [21]. However, these approaches remain in development and further attention is needed on integration with EHR systems and appropriate anonymization [22]. This discussion on disease definitions may also apply to treatments, where a similar process for extraction of coded data may be relevant to the research question.

Outcome definitions

Outcome data can also be extracted from the EHR, although historically there has been a lack of transparency in published articles about the source of these critical data. Deriving outcomes from

routine clinical practice has distinct advantages, including cost and broad generalisability. However, the validity, timeliness and appropriateness of data need careful examination if results are to affect clinical decisions. Similarly, detail on the coding systems and individual codes utilised for each outcome are essential for interpretation and subsequent confirmation of results [23].

Consensus on which study endpoints can be accurately collected using coded EHR data could help better focus these efforts. Mortality is one area where many countries have robust database systems (although tracking and recording still vary considerably). Other outcomes are unlikely to be recorded to the same level of precision, for example subtypes of myocardial infarction [24]. Concordance between primary and secondary care can be high in healthcare settings with good linkage [25]. Concordance between hospital discharge codes and physician review of medical records depends on the endpoint, with agreement substantial for myocardial infarction, deep venous thrombosis, stroke and pulmonary embolism (kappa 0.78-0.84), even higher for coronary bypass surgery (kappa 0.92), but much lower for peripheral vascular disease, angina and congestive heart failure (kappa 0.40-0.56) [26].

Two analyses from randomised trials highlight that the information gained using structured healthcare data may differ from what we might expect from more traditional adjudication endpoint committees [27]. However, they also show that the output is no less valid and could herald, if used appropriately, a new era of large-scale trials embedded within routine healthcare at much lower cost and with virtually indefinite follow-up. In a randomised trial of clarithromycin in patients with ischaemic heart disease, there was only modest agreement between outcomes from the formal adjudication committee compared to using routine healthcare data. However, this had no appreciable impact on the estimated intervention effect [28]. For the primary outcome of all-cause mortality, myocardial infarction or unstable angina, the hazard ratio was 1.15 using adjudication (95% CI 0.99-1.34), versus 1.13 using the public register data (95% CI 0.98-1.30). Similarly, in a randomised trial looking at the effects of aspirin and of omega-3 fatty acids in patients with diabetes (ASCEND), rate ratios for vascular events for both interventions were identical when comparing adjudicated with EHR follow-up [29, 30]. The value of trials based on routinely collected data may vary according to the clinical topic under study [31].

New perspectives for enhanced use of EHR data

The advent of more structured EHR systems allows for a range of applications to enhance the value of routine data, including neural network and artificial intelligence (AI) approaches for participant selection and analytics. Predictive modelling is anticipated to drive improvements in healthcare and personalised medicine [32]. Challenges include the availability and access to data, consent, privacy and data security. There are many appropriate barriers to sharing data, although the coronavirus pandemic has highlighted the need for open datasets for AI forecasting alongside the responsible use of data and digital health tools such as contact tracing. Better approaches to achieve anonymisation/pseudonymisation of data and federated learning are being developed. Data standardisation and interoperability (including semantic interoperability) across countries and regions is a major limitation, but one that international groups are specifically targeting. Prominent retractions of COVID-19 studies using the Surgisphere registry [33, 34] reinforce the need for complete transparency and traceability of published data and public accountability for data quality.

Randomized trials remain highly labour intensive, and the use of EHR data presents an opportunity to radically change our approach to healthcare-embedded evidence generation. In the aforementioned ASCEND trial, the coordinating centre sent 250,000 follow-up forms by post,

10,000 web-based forms, 10,000 mailings to physicians, and 60,000 telephone calls [35]. In addition, RCTs face considerable regulatory, contractual, administrative and financial burdens leading to poor success rates for completion. Two distinct approaches have been developed in recent years to make use of EHR data for RCTs – the registry-based trial and true EHR-embedded trials. The common thread is that mass pre-screening can occur to support large pragmatic clinical trials, enhancing recruitment and providing patient follow-up.

The Thrombus Aspiration in ST-Elevation Myocardial Infarction in Scandinavia (TASTE) trial is a prime example of a multicentre RCT using the infrastructure of a population-based registry. Randomising 7259 patients, the trial results changed routine practice (in this case, showing that thrombus aspiration during a myocardial infarction did not reduce 30-day mortality compared to coronary stenting alone) [36]. At that point in time, thrombus aspiration was routinely performed in clinical practice due to favourable results from smaller or industry-funded studies. This registry-RCT approach was therefore critical to be able to randomise a large enough population to alter clinical pathways. Consortia such as EuroHeart by the European Society of Cardiology are working towards synergising registry infrastructure across international borders to enable registry-based RCTs at even greater scale [37]. Currently in its pilot phase, EuroHeart is setting up infrastructure to establish platforms for countries to develop their own cardiology registries, and to collect a common dataset to facilitate cross-country studies.

An alternative approach is to run trials directly or indirectly through the EHR, both for defining the patient population as well as ascertaining outcomes. In the UK, 1 in 4 primary care centres are part of the Clinical Practice Research Datalink, an agency of the UK medicines regulator, allowing for pseudonymised pre-screening of over 15 million patients. The DaRe2 approach (healthcare Data for pragmatic clinical Research in the NHS – primary 2 secondary) is utilising this system to perform a "remote-RCT" (DaRe2THINK randomised trial with no physical patient contact required), with the ability to link all primary and secondary care coded health data [23]. The trial aims to ascertain the efficacy and cost-effectiveness of giving younger patients with atrial fibrillation direct oral anticoagulants to prevent thromboembolism and cognitive decline [38]. Hospitalisation records can also be used for participant identification, such as the ORION-4 trial investigating inclisiran for LDL lowering [39].

For both registry and EHR-based trial approaches, the major international limitation is the ability to perform such studies, currently restricted to countries with linked national public healthcare systems. Where these types of trials are possible, they open up new avenues for cost-efficiency and a low burden for clinical staff and participants.

Current approaches for assimilating EHR-based evidence

The key challenges and opportunities for coded healthcare data in the context of research are outlined in **Figure 1** (main article), which presents the life-cycle of EHR coding from initial notation to their potential use to enhance clinical practice.

Publication process

Scientific journals have taken steps to increase transparency, including data sharing statements and publication of software code. However, journals do not currently have sufficient mechanisms in place to ensure these processes are actually followed through after publication, or to apply remedial action. Reporting checklists are commonly required by all major journals, asking authors to state if

certain critical items are reported in their paper, but there are no consequences, for example, if authors do not pre-publish their design or code lists. Despite checklists improving reporting, there is still evidence of substantial suboptimal practice.[40, 41] For AI-based articles, the SPIRIT-AI Extension includes 15 new items [42], an initiative started after a meta-analysis of AI-based imaging studies found very few with externally validated results.[43]

Regulatory and health technology assessment requirements

Real-world data offers exciting opportunities to assist in rapid drug and device approvals by regulators, but there remains considerable concern as to how regulators can ensure that data is of sufficient quality, and that the rights of patients are protected. The post-marketing space is also an area of opportunity for regulatory decision-making, for example the US Food and Drug Administration (FDA) Breakthrough Device Program [44]. A breakthrough device has a reasonable expectation of providing more effective treatment or diagnosis of a life-threatening or debilitating condition, with intermediate or surrogate endpoints sufficient for device approval (for example, quality of life or the six-minute walk test in heart failure). The FDA may accept a greater extent of uncertainty of the benefit-risk profile for these devices if appropriate under the circumstances, including that the uncertainty is sufficiently balanced by other factors such as adequate post-market controls to support pre-market approval. The National Evaluation System for health Technology Coordinating Center (NESTcc) project is a collaboration between the Medical Device Innovation Consortium, FDA and other stakeholders, aiming to develop frameworks for evaluating real-world evidence for use in clinical and regulatory decisions. The NESTcc methods [45] and data quality framework [45] provide an opportunity to generate test cases to support regulatory decisions.

In Europe, the Heads of Medicines Agencies and European Medicines Agency (EMA) joint Big Data Task Force published recommendations in 2020 aimed at closing the gap between knowledge of data quality and risk evaluation of medicines [46]. Among its recommendations are to establish a platform, the Data Analysis and Real World Interrogation Network (DARWIN), to access and analyse healthcare data from across the EU. Other goals are to develop frameworks for data quality, collaboration and data discoverability. A qualification process now exists for large patient registry platforms, in which scientists must demonstrate to EMA the quality of the data, how the quality of data entry is audited, and the methods of analysis.

In summary, there are a number of opportunities for EHR studies to support drug and device development, including access to large patient numbers using therapy in clinical practice, the potential for unbiased follow-up for trials that lack blinding or a placebo arm, and near indefinite follow-up to monitor safety and durability of effect. These positives must be balanced against the limitations of different EHR systems, inequity across regions and variability in quality standards, data security and governance.

Implementation to clinical practice

Big data research can support the generation of clinical practice guidelines. The Grading of Recommendations Assessment, Development and Evaluation (GRADE) system [47] can assist in rating the quality of evidence of various sources, although at present many task forces may lack the expertise to appropriately review and contextualize the evidence from EHR and registry sources. A clear potential from big data analysis is to: (1) Provide more contemporary evidence, updating policies that are often based on dated studies that do not reflect current disease burden [48]; and (2) Address multimorbidity, whereas historical clinical practice guidelines are typically based on trials where patients have a much lower rate of multimorbidity than the population at-risk.

Patient concerns and privacy

Social License

Lack of public trust is detrimental to the goals of data sharing. Legal compliance with data protection and privacy requirements does not necessarily provide social legitimacy. Research suggests widespread, but conditional, support from patients and the public for sharing their health data [49], with concerns about abuse of data and commercial use (for example passive transmission of smartphone data). A European Heart Network paper on the value of digital technologies triggered a series of exchanges with patients and found general support for digital tools and sharing of data to improve health outcomes, provided there is an ethical framework [50]. A review addressing the views and attitudes of patients and the public towards the use of health data for research also found a supportive public attitude; however, they expressed a need to trust researchers to be transparent and responsible, and to make an effort to avoid abuse, be accountable and mitigate risks in health research [51].

The concept of a social licence has now been drawn into the health data domain [52], and refers to acceptance by the public and other stakeholders, often beyond pure legal compliance. A social license has the potential to improve and stimulate big data projects. It would steer the practice of health data sharing towards more responsibility and sustainability, whilst respecting current rules and regulations, and lessening administrative burden. A social licence also fulfils a communication and informal educational need for practitioners, patients and the public. Early engagement in the research life-cycle opens up learning possibilities for researchers to better understand what patients and the public want, and conversely for the public to understand what can be achieved by analysis of routine health data. Co-creation and co-design could lead to a stronger basis for big data health research, as well as 'fit-for-purpose' output.

Public mandate

Public concerns about data sharing include the potential for breaches in confidentiality, uncertainty on who has access to personal data, the impact of inappropriate access, and the use of data for commercial purposes. For example, the UK National Institute for Health and Care Excellence (NICE) Citizens Council expressed concerns about the potential for data to be sold to other organisations and used for profit or for purposes other than research.[53] This suggests the need to look beyond compliance and move towards a governance system that maximises the liberties of research to serve the public and the common good for health protection and health improvement, but restricts potential abuse of data.

There are a number of challenges that must be addressed before a sustainable mandate can be obtained from the public and patients to use, share and reuse their health data. One is to reverse the mindset that governance is a top-down activity, where patients and the public are viewed as "data subjects" needing to be nudged into participating in research. Another challenge is the status of research as a common good, whereas large consortia and big data projects have a multitude of research goals, some of which transcend pure public service and may not be aligned with the public's concept of the unmet need. Finally, reciprocity between the contributions of patients/public and researchers, and the insufficient attention paid to generation of widely usable and accessible products. Above all, clinical research can be conducted so that participants are aware of how their data can be used by other researchers, and that provision of anonymised data can enhance global scientific progress.

Informed consent

Informed consent from individuals is typically a requirement prior to their involvement in research, but how this applies in the context of very large-scale data (for example within an EHR) remains challenging. Where personal data can be anonymised or pseudonymised, human research ethics committees supported by legislation have tended to allow research without individual consent, where this is for the public good. The purpose often needs to be defined together with the data controllers, data processors and the technical method for using the data. For primary use, where data are collected from millions of patients rather than hundreds or thousands, obtaining consent may be impossible. The use of data 'opt outs' is common practice, but still requires an initial explanation of purpose when data are first collected. Secondary use is more challenging, since reaching individual patients to consent for new research may not be possible. Integrated consent models may provide a solution, for example within registries. Where anonymised and aggregated data are used, adequately informing patients of the person/group-level unit of analysis, plus a guarantee that their data cannot be traced or linked by third parties is important. Where consent is possible, there is often a lack of clarity on what exactly is being agreed to and how consent can be revoked. This raises the possibility that consent may be both time-limited and impact-limited, the so-called dynamic informed consent model [54].

Patient and Public involvement

Involving representatives of the public as well as patients with the disease of interest can be a powerful method to achieve research that addresses the concerns and needs of patients [55, 56], while also taking account of specific issues regarding the use of EHR data. Although some research funders mandate public involvement in the design and management of studies, internationally this remains an evolving field despite the potential advantages within data-intensive health research [57]. **Figure 2** (main article) describes a potential structure for engagement of the public that can constructively benefit both big data research and the public.

References

1. U.S. Food & Drug Administration. Submitting documents using real-world data and real-world evidence to fda for drugs and biologics guidance for industry. *FDA-2019-D-1263 Guidance Document.* 2019: https://www.fda.gov/media/124795/download [Accessed 19 Jul 2021].

2. A Sammani, A Bagheri, PGM van der Heijden, A Te Riele, AF Baas, CAJ Oosters, D Oberski, FW Asselbergs. Automatic multilabel detection of ICD10 codes in Dutch cardiology discharge letters using neural networks. *NPJ Digit Med.* 2021;4:37.

3. MC Blom, M Khalid, B Van-Lettow, H Hutink, S Larsson, S Huff, M Ingvar. Harmonization of the ICHOM Quality Measures to Enable Health Outcomes Measurement in Multimorbid Patients. *Front Dig Health.* 2020;2: 10.3389/fdgth.2020.606246.

4. DA Springate, E Kontopantelis, DM Ashcroft, I Olier, R Parisi, E Chamapiwa, D Reeves. ClinicalCodes: an online clinical codes repository to improve the validity and reproducibility of research using electronic medical records. *PLoS One.* 2014;9:e99825.

5. K Mahbubani, F Georgiades, EL Goh, S Chidambaram, P Sivakumaran, T Rawson, S Ray, A Hudovsky, D Gill. Clinician-directed improvement in the accuracy of hospital clinical coding. *Future Healthc J.* 2018;5:47-51.

6. R Farmer, R Mathur, K Bhaskaran, SV Eastwood, N Chaturvedi, L Smeeth. Promises and pitfalls of electronic health record analysis. *Diabetologia.* 2018;61:1241-8.

7. K Lucyk, K Tang, H Quan. Barriers to data quality resulting from the process of coding health information to administrative data: a qualitative study. *BMC Health Serv Res.* 2017;17:766.

8. CG Chute. Coding patient information, reimbursement for care, and the ICD transition. *Virtual Mentor.* 2013;15:596-9.

9. EM Burns, E Rigby, R Mamidanna, A Bottle, P Aylin, P Ziprin, OD Faiz. Systematic review of discharge coding accuracy. *J Public Health (Oxf).* 2012;34:138-48.

10. MR Cowie, JI Blomster, LH Curtis, et al. Electronic health records to facilitate clinical research. *Clin Res Cardiol.* 2017;106:1-9.

11. MA Bohensky, D Jolley, V Sundararajan, S Evans, DV Pilcher, I Scott, CA Brand. Data linkage: a powerful research tool with potential problems. *BMC Health Serv Res.* 2010;10:346.

12. K Harron, C Dibben, J Boyd, A Hjern, M Azimaee, ML Barreto, H Goldstein. Challenges in administrative data linkage for research. *Big Data Soc.* 2017;4:2053951717745678.

13. Observational Medical Outcomes Partnership. OMOP Common Data Model. Vol 2020.

14. MD Wilkinson, M Dumontier, IJ Aalbersberg, et al. The FAIR Guiding Principles for scientific data management and stewardship. *Sci Data.* 2016;3:160018.

15. S Denaxas, A Gonzalez-Izquierdo, K Direk, et al. UK phenomics platform for developing and validating electronic health record phenotypes: CALIBER. *J Am Med Inform Assoc.* 2019;26:1545-59.

16. H Mo, WK Thompson, LV Rasmussen, et al. Desiderata for computable representations of electronic health records-driven phenotype algorithms. *J Am Med Inform Assoc.* 2015;22:1220-30.

17. V Kuan, S Denaxas, A Gonzalez-Izquierdo, et al. A chronological map of 308 physical and mental health conditions from 4 million individuals in the English National Health Service. *Lancet Digit Health.* 2019;1:e63-e77.

18. JC Kirby, P Speltz, LV Rasmussen, et al. PheKB: a catalog and workflow for creating electronic phenotype algorithms for transportability. *J Am Med Inform Assoc.* 2016;23:1046-52.

19. DR Morales, MM Conover, SC You, et al. Renin-angiotensin system blockers and susceptibility to COVID-19: a multinational open science cohort study. *medRxiv.* 2020, 10.1101/2020.06.11.20125849.

20. MJ Schuemie, PB Ryan, N Pratt, R Chen, SC You, HM Krumholz, D Madigan, G Hripcsak, MA Suchard. Principles of Large-scale Evidence Generation and Evaluation across a Network of Databases (LEGEND). *J Am Med Inform Assoc.* 2020;27:1331-7.

21. LT Slater, W Bradlow, S Ball, R Hoehndorf, GV Gkoutos. Improved characterisation of clinical text through ontology-based vocabulary expansion. *J Biomed Semantics.* 2021;12:7.

22. S Sheikhalishahi, R Miotto, JT Dudley, A Lavelli, F Rinaldi, V Osmani. Natural Language Processing of Clinical Notes on Chronic Diseases: Systematic Review. *JMIR Med Inform.* 2019;7:e12239.
23. X Wang, AR Mobley, O Tica, et al. Systematic approach to outcome assessment from coded electronic healthcare records in the DaRe2THINK NHS-embedded randomised trial. *medRxiv.* 2022: https://doi.org/10.1101/2022.05.24.22275434 [Accessed: 24 Jun 2022].
24. C Held. When do we need clinical endpoint adjudication in clinical trials? *Ups J Med Sci.* 2019;124:42-5.
25. R Persson, T Sponholtz, C Vasilakis-Scaramozza, KW Hagberg, T Williams, D Kotecha, P Myles, SS Jick. Quality and Completeness of Myocardial Infarction Recording in Clinical Practice Research Datalink Aurum. *Clin Epidemiol.* 2021;13:745-53.
26. SR Heckbert, C Kooperberg, MM Safford, BM Psaty, J Hsia, A McTiernan, JM Gaziano, WH Frishman, JD Curb. Comparison of self-report, hospital discharge codes, and adjudication of cardiovascular events in the Women's Health Initiative. *Am J Epidemiol.* 2004;160:1152-8.
27. JM Franklin, E Patorno, RJ Desai, et al. Emulating Randomized Clinical Trials With Nonrandomized Real-World Evidence Studies: First Results From the RCT DUPLICATE Initiative. *Circulation.* 2021;143:1002-13.
28. E Kjoller, J Hilden, P Winkel, et al. Agreement between public register and adjudication committee outcome in a cardiovascular randomized clinical trial. *Am Heart J.* 2014;168:197-204 e1-4.
29. L Bowman, M Mafham, K Wallendszus, et al. Effects of Aspirin for Primary Prevention in Persons with Diabetes Mellitus. *N Engl J Med.* 2018;379:1529-39.
30. L Bowman, M Mafham, K Wallendszus, et al. Effects of n-3 Fatty Acid Supplements in Diabetes Mellitus. *N Engl J Med.* 2018;379:1540-50.
31. KA Mc Cord, H Ewald, A Agarwal, D Glinz, S Aghlmandi, JPA Ioannidis, LG Hemkens. Treatment effects in randomised trials using routinely collected data for outcome assessment versus traditional trials: meta-research study. *Bmj.* 2021;372:n450.
32. A Karwath, KV Bunting, SK Gill, et al. Redefining beta-blocker response in heart failure patients with sinus rhythm and atrial fibrillation: a machine learning cluster analysis. *Lancet.* 2021;398:1427-35.
33. MR Mehra, SS Desai, S Kuy, TD Henry, AN Patel. Retraction: Cardiovascular Disease, Drug Therapy, and Mortality in Covid-19. N Engl J Med. DOI: 10.1056/NEJMoa2007621. *N Engl J Med.* 2020;382:2582.
34. The Editors of the Lancet Group. Learning from a retraction. *The Lancet.* 2020;396:1056.
35. L Bowman, M Mafham, W Stevens, R Haynes, T Aung, F Chen, G Buck, R Collins, J Armitage. ASCEND: A Study of Cardiovascular Events iN Diabetes: Characteristics of a randomized trial of aspirin and of omega-3 fatty acid supplementation in 15,480 people with diabetes. *Am Heart J.* 2018;198:135-44.
36. O Frobert, B Lagerqvist, GK Olivecrona, et al. Thrombus aspiration during ST-segment elevation myocardial infarction. *N Engl J Med.* 2013;369:1587-97.
37. L Wallentin, CP Gale, A Maggioni, I Bardinet, B Casadei. EuroHeart: European Unified Registries On Heart Care Evaluation and Randomized Trials. *Eur Heart J.* 2019;40:2745-9.
38. D Kotecha, D Shukla, Clinical Practice Research Datalink. Preventing stroke, premature death and cognitive decline in a broader community of patients with atrial fibrillation using healthcare data for pragmatic research: A randomised controlled trial (DaRe2THINK). *Sponsor: University of Birmingham; Funder: National Institute for Health Research.* 2021;EudraCT 2020-005774-10; ClinicalTrials.gov NCT04700826: https://www.birmingham.ac.uk/dare2think [Accessed 30 Mar 2022].
39. L Bowman. A Randomized Trial Assessing the Effects of Inclisiran on Clinical Outcomes Among People With Cardiovascular Disease (ORION-4). *Sponsor: University of Oxford; Funder: Novartis Pharmaceuticals.* 2018;ClinicalTrials.gov NCT03705234: https://www.orion4trial.org/.

40. ZW Tan, AC Tan, T Li, et al. Has the reporting quality of published randomised controlled trial protocols improved since the SPIRIT statement? A methodological study. *BMJ Open.* 2020;10:e038283.
41. L Turner, L Shamseer, DG Altman, et al. Consolidated standards of reporting trials (CONSORT) and the completeness of reporting of randomised controlled trials (RCTs) published in medical journals. *Cochrane Database Syst Rev.* 2012;11:Mr000030.
42. SC Rivera, X Liu, AW Chan, AK Denniston, MJ Calvert, AI Spirit, Consort-AI Working Group. Guidelines for clinical trial protocols for interventions involving artificial intelligence: the SPIRIT-AI Extension. *BMJ.* 2020;370:m3210.
43. X Liu, L Faes, AU Kale, et al. A comparison of deep learning performance against health-care professionals in detecting diseases from medical imaging: a systematic review and meta-analysis. *Lancet Digit Health.* 2019;1:e271-e97.
44. US Food and Drug Administration. Breakthrough Devices Program: Guidance for Industry and Food and Drug Administration Staff. 2018: https://www.fda.gov/media/108135/download [Accessed: 16 Sep 2021].
45. National Evaluation System for health Technology Coordinating Center. NESTcc Methods Framework. 2020: https://nestcc.org/nestcc-methods-framework/ [Accessed 16 Sep 2021].
46. HMA-EMA Joint Big Data Taskforce. Summary report EMA/105321/2019. *https://www.hma.eu/fileadmin/dateien/HMA_joint/00-_About_HMA/03-Working_Groups/Big_Data/2019_02_HMAEMA_Joint_Big_DataTaskforce_summary_report.pdf.* 2019.
47. GH Guyatt, AD Oxman, GE Vist, R Kunz, Y Falck-Ytter, P Alonso-Coello, HJ Schünemann. GRADE: an emerging consensus on rating quality of evidence and strength of recommendations. *BMJ.* 2008;336:924-6.
48. OJ Ziff, M Samra, JP Howard, DI Bromage, F Ruschitzka, DP Francis, D Kotecha. Beta-blocker efficacy across different cardiovascular indications: an umbrella review and meta-analytic assessment. *BMC Med.* 2020;18:103.
49. M Aitken, S Cunningham-Burley, C Pagliari. Moving from trust to trustworthiness: Experiences of public engagement in the Scottish Health Informatics Programme. *Sci Public Policy.* 2016;43:713-23.
50. Scherrenberg M, Vangenechten G, Janssen A, Dendale P. What is the value of digital tools for cardiovascular patients? . *European Heart Network.* 2020: http://www.ehnheart.org/publications-and-papers/publications/1285:digital-tools-cardiovascular-patients.html [Accessed: 16 Sep 2021].
51. S Kalkman, J van Delden, A Banerjee, B Tyl, M Mostert, G van Thiel. Patients' and public views and attitudes towards the sharing of health data for research: a narrative review of the empirical evidence. *J Med Ethics.* 2019, 10.1136/medethics-2019-105651.
52. P Carter, GT Laurie, M Dixon-Woods. The social licence for research: why care.data ran into trouble. *J Med Ethics.* 2015;41:404-9.
53. NICE Citizens Council Reports. *What Ethical and Practical Issues Need to Be Considered in the Use of Anonymised Information Derived from Personal Care Records as Part of the Evaluation of Treatments and Delivery of Care?* London: National Institute for Health and Care Excellence (NICE); 2015.
54. J Kaye, EA Whitley, D Lund, M Morrison, H Teare, K Melham. Dynamic consent: a patient interface for twenty-first century research networks. *Eur J Hum Genet.* 2015;23:141-6.
55. JC Crocker, I Ricci-Cabello, A Parker, JA Hirst, A Chant, S Petit-Zeman, D Evans, S Rees. Impact of patient and public involvement on enrolment and retention in clinical trials: systematic review and meta-analysis. *BMJ.* 2018;363:k4738.
56. KV Bunting, M Stanbury, O Tica, D Kotecha. Transforming clinical research by involving and empowering patients- the RATE-AF randomized trial. *Eur Heart J.* 2021;42:2411-4.
57. M Aitken, MP Tully, C Porteous, et al. Consensus Statement on Public Involvement and Engagement with Data-Intensive Health Research. *Int J Pop Data Sci.* 2019;4:06.
