# Supplementary material for: CODE-EHR best practice framework for the use of structured electronic healthcare records in clinical research
Source: BMJ. 2022 Aug 29;378:e069048. doi: 10.1136/bmj-2021-069048 (PMC9403753; doi:10.1136/bmj-2021-069048)
Supplement: Supplementary file 2 — Web appendix 2: Using the CODE-EHR reporting checklist [file kotd069048.ww2.pdf]

CODE-EHR best practice framework for the use of structured electronic healthcare records in clinical research

Appendix 2: Using the CODE-EHR reporting checklist

The following text provides best practice examples for completing each domain of the CODE-EHR framework.

1. Dataset construction and linkage

Objective	Framework standards
To provide an understanding of how the structured healthcare data were identified and used.	Minimum: Flow diagram of datasets used in the study, and description of the processes and directionality of any linkage performed, published within the research report or supplementary documents.<br>Preferred: Provided within a pre-published protocol or open-access document.

(a) State the source of any datasets used.

This section describes the EHR system and data extract, transform and load (ETL) tools that are used to construct the dataset. In addition, source details can be provided of the individual datasets that are linked, such as national registry data, primary care data, national laboratory COVID-19 testing data, or other local non-EHR data sources such as imaging, electrocardiography, etc. A flow diagram to illustrate the data extraction process could be helpful for this item.

(b) Comment on how the observed and any missing data were identified and addressed, and the proportion observed for each variable.

Besides describing both observed and unobserved data, and listing the proportion of missing data for each variable, this item addresses how missing data were handled, whether imputation was performed and what methods were used.

(c) Provide data on completeness of follow-up.

This item focuses on the description of follow-up and censoring of patients, whether other sources were used to complete follow-up beyond the EHR, and how discrepancies are managed between the different data sources.

(d) For linked datasets, specify how linkage was performed and the quality of linkage methods.

The identifier and methods (e.g. probabilistic or deterministic) used to link the different data sources and any prior work assessing the quality of linkage are discussed in this item. A worked example is the recently published description of the England-wide electronic health record resource to investigate the relation between COVID-19 and cardiovascular disease [1]. In addition, use of common data models to link different datasets can be described such as the Observational Medical Outcomes Partnership (OMOP) common data model (www.ohdsi.org/) or Fast Healthcare Interoperability Resources (www.hl7.org/fhir/).

2. Data fit for purpose

Objective	Framework standards
To ensure transparency with the approach taken, with respect to coding of the structured healthcare data.	Minimum: Clear unambiguous statements on the process of coding in the methods section of the research report.<br>Preferred: Provided within a pre-published protocol or open-access document.

(a) Confirm origin, clinical processes, and the purpose of data

This section addresses whether coding was done by administrators or healthcare professionals and whether this was done for care, research, auditing or reimbursement purposes. In particular, whether financial incentives are in place that may influence data entry and/or explain potential heterogeneity or bias when comparing data across institutes and healthcare systems. Furthermore, the workflow when data entry was performed in the clinical process can be explained; for example, retrospectively based on the discharge letter or at the start of a diagnostic pathway.

(b) Specify coding systems, clinical terminologies or classifications used and their versions, and any manipulation of the data.

This item focuses on which clinical terminologies or classification systems are used during the study period. Examples are the International Classification of Diseases code (ICD), Current Procedure Terminology codes (CPT) or OPCS Classification of Interventions and Procedures that are commonly used for billing; or the Systematized Nomenclature of Medicine-Clinical Terms (SNOMED-CT) which is a standardized clinical terminology. Other coding systems such as the Anatomical Therapeutic Chemical code (ATC) for medicine can be listed in this item. The specific versions of the system used need to be specified.

(c) Provide detail on quality assessment for data capture.

This item provides an opportunity to disclose any metrics on quality assessment in terms of data capture, either from the current study or prior publications/reviews. Assessment of any amendments that were made when extracting data from EHR systems can be addressed, such as outlier filters or imputation of missing data.

(d) Outline potential sources of bias.

Potential biases can occur when merging structured healthcare data from different settings (e.g. primary care and hospital records), different institutes and healthcare systems. Differences in billing across healthcare systems, but also availability of tests and procedures can influence results that may need discussion. Another potential source of bias can be caused by the use of different consent types across sites. Age, sex and socioeconomic status can influence participation in observational studies that may have consequences for the generalisability of the results [2].

3. Disease and outcome definitions

Objective	Framework standards
To fully detail how conditions AND outcome events were defined, allowing other researchers to identify errors and repeat the process in other datasets.	Minimum: State what codes were used to define diseases, treatments, conditions and outcomes *prior to statistical analysis*, including those relating to patient identification, therapy, procedures, comorbidities, and components of any composite endpoints.<br>Preferred: Provided within a pre-published protocol or open-access document *prior to statistical analysis.*

(a) Detailed lists of codes used for each aspect of the study.

This includes all the disease codes or definitions across the different data sources, covering variables for selection criteria, baseline characteristics, medications, therapies and interventions, and any safety or outcome events. For EHR data, an example is the primary and secondary healthcare coding used for the DaRe2THINK clinical trial [3]; and for registry data, the publication of agreed data standards and definitions by EuroHeart [4]. Preferably, the phenotypes are defined before the statistical analysis phase and made available in an open-access repository, such as the HDR-UK Phenotype library (https://phenotypes.healthdatagateway.org) or the Phenotype KnowledgeBase (https://phekb.org).

(b) Date of publication and access details for the coding manual.

Details regarding date and access can be provided in this section for the phenotype and outcomes library including the individual coding systems. For a controlled trial, it is advisable that disease and outcome definitions are published prior to any interim or final data analysis.

(c) Provide definitions, implementation logic and validation of any phenotyping algorithms used.

Description of how phenotypes were generated (i.e. how conditions and outcome events were defined), allowing other researchers to replicate results in other datasets and further improve any phenotyping algorithms.

(d) Specify any processes used to validate the coding scheme or reference to prior work.

Previous or published work can be listed to support the use or re-use of the proposed disease and outcome definitions. Lack of prior validation work for coding schema, or validation work in-progress can be specifically mentioned.

4. Analysis

Objective	Framework standards
To fully detail how outcome events were analysed and allow independent assessment of the authenticity of study findings.	Minimum: Describe the process used to analyse study outcomes, including statistical methods and use of any machine learning or algorithmic approaches.<br>Preferred: Provide a statistical analysis plan as a supplementary file, locked *prior to analyses commencing.*

(a) Provide details on all statistical methods used.

The statistical methods are preferably pre-published in an open access repository together with the protocol, including the clinical terminologies and classification systems.

(b) Provide links to any machine code or algorithms used in the analysis, preferably as open-source.

A link to open access repositories such as the source code management system GitHub is advised, *depending on Intellectual Property restrictions*. For example, in a recent study from the CVD-COVID-UK/COVID-IMPACT consortium [5], the following paragraph was added to the methods section :

> "Information on the data used can be found on the HDR UK Gateway https://web.www.healthdatagateway.org/dataset/7e5f0247-f033-4f98-aed3-3d7422b9dc6d; EHR phenotyping algorithms can be downloaded in machine-readable formats from the HDR UK Phenotype Library http://phenotypes.healthdatagateway.org. All code and phenotypes are available at github.com/BHFDSC/CCU002_02."

(c) Specify the processes of testing assumptions, assessing model fit and any internal validation.

This item describes the process of internal validation (quantifying the performance of the model in the given population). For example, a prediction tool developed for cardiovascular disease in patients with type 2 diabetes used split sample validation, and justified their approach clearly in the supplementary appendix [6].

(d) Specify how generalisability of results was assessed, the replication of findings in other datasets, or any external validation.

External validation assesses generalisability of results (often by testing models in other related populations). A clear description of the external dataset is needed in comparison to the original dataset. Limitations can be discussed regarding the applicability of the developed algorithm and to identify any gaps for future validation efforts by others. In particular, this could consider potential divergence according to ethnicity, social-economic status, severity of disease, age or sex. EHRs can be a valuable tool for external validation of prediction models, as previously discussed [7].

5. Ethics and governance

Objective	Framework standards
To provide patients, who may or may not have given consent, and regulatory authorities the ability to interrogate the security and providence of the data.	Minimum: Clear unambiguous statements on how the principles of Good Clinical Practice and Data Protection will be/were met, provided in the methods section of the research report.<br><br>Preferred: Provided within a pre-published protocol or open-access document with evidence of patient and public engagement.

(a) State how informed consent was acquired, or governance if no patient consent.

Specify whether waiver of consent, opt-out or opt-in was used for the study. Describe how information governance was arranged if no explicit consent was obtained.

(b) Specify how data privacy was protected in the collection and storage of data.

This item describes who accessed the original data and how the de-identification process was managed. For studies using text mining of EHRs and natural language processing, additional detail on the steps taken for anonymisation and its verification are required.

(c) Detail what steps were taken for patient and public involvement in the research study.

It is advised to involve patient and public representatives as partners throughout the research project, and critical in cases where consent was waived. A description is helpful of how patients and the public were involved, including details on whether patients and the public were part of the decision-making regarding the design and objectives of the study, the definition of phenotypes, outcomes and the chosen dissemination strategy [8].

(d) Provide information on where anonymised source data or code can be obtained for verification and further research.

This could include the actual datasets used in the study, or clear instruction on how researchers can interact with the study team or data providers to obtain access for additional research. Datasets can be published in specialised journals, such as the Scientific Data Journal from the Nature group (https://www.nature.com/sdata/). Procedures to access data can be described as done by the UK Biobank (https://www.ukbiobank.ac.uk/), the Medical Information Mart for Intensive Care dataset from Beth Israel Deaconess Medical Center (https://mimic.mit.edu), or UK primary care data by the Clinical Practice Research Datalink (https://cprd.com/).

References

1. A Wood, R Denholm, S Hollings, J Cooper, S Ip, V Walker, S Denaxas, A Akbari, A Banerjee, W Whiteley, A Lai, J Sterne, C Sudlow, C-C-U consortium. Linked electronic health records for research on a nationwide cohort of more than 54 million people in England: data resource. *BMJ.* 2021;373:n826.

2. ME Kho, M Duffett, DJ Willison, DJ Cook, MC Brouwers. Written informed consent and selection bias in observational studies using medical records: systematic review. *BMJ.* 2009;338:b866.

3. X Wang, AR Mobley, O Tica, K Okoth, RE Ghosh, P Myles, T Williams, S Haynes, K Nirantharakumar, D Shukla, D Kotecha. Systematic approach to outcome assessment from coded electronic healthcare records in the DaRe2THINK NHS-embedded randomised trial. *medRxiv.* 2022: https://doi.org/10.1101/2022.05.24.22275434 [Accessed: 24 Jun 2022].

4. G Batra, S Aktaa, L Wallentin, AP Maggioni, P Ludman, D Erlinge, B Casadei, CP Gale. Data standards for acute coronary syndrome and percutaneous coronary intervention: the European Unified Registries for Heart Care Evaluation and Randomised Trials (EuroHeart). *Eur Heart J.* 2022, 10.1093/eurheartj/ehac133.

5. WN Whiteley, S Ip, JA Cooper, T Bolton, S Keene, V Walker, R Denholm, A Akbari, E Omigie, S Hollings, E Di Angelantonio, S Denaxas, A Wood, JAC Sterne, C Sudlow, C-C-U consortium. Association of COVID-19 vaccines ChAdOx1 and BNT162b2 with major venous, arterial, or thrombocytopenic events: A population-based cohort study of 46 million adults in England. *PLoS Med.* 2022;19:e1003926.

6. GFN Berkelmans, S Gudbjornsdottir, FLJ Visseren, SH Wild, S Franzen, J Chalmers, BR Davis, NR Poulter, AM Spijkerman, M Woodward, SL Pressel, AK Gupta, YT van der Schouw, AM Svensson, Y van der Graaf, SH Read, B Eliasson, JAN Dorresteijn. Prediction of individual life-years gained without cardiovascular events from lipid, blood pressure, glucose, and aspirin treatment based on data of more than 500 000 patients with Type 2 diabetes mellitus. *Eur Heart J.* 2019;40:2899-906.

7. RD Riley, J Ensor, KI Snell, TP Debray, DG Altman, KG Moons, GS Collins. External validation of clinical prediction models using big datasets from e-health records or IPD meta-analysis: opportunities and challenges. *BMJ.* 2016;353:i3140.

8. KV Bunting, M Stanbury, O Tica, D Kotecha. Transforming clinical research by involving and empowering patients- the RATE-AF randomized trial. *Eur Heart J.* 2021;42:2411-4.
