# Supplementary material for: CODE-EHR best practice framework for the use of structured electronic healthcare records in clinical research
Source: BMJ. 2022 Aug 29;378:e069048. doi: 10.1136/bmj-2021-069048 (PMC9403753; doi:10.1136/bmj-2021-069048)
Supplement: Supplementary file 3 — Web appendix 3: Derivation of the CODE-EHR framework [file kotd069048.ww3.pdf]

CODE-EHR best practice framework for the use of structured electronic healthcare records in clinical research

Appendix 3: Derivation of the CODE-EHR framework

A) Summary flowchart of stakeholder events

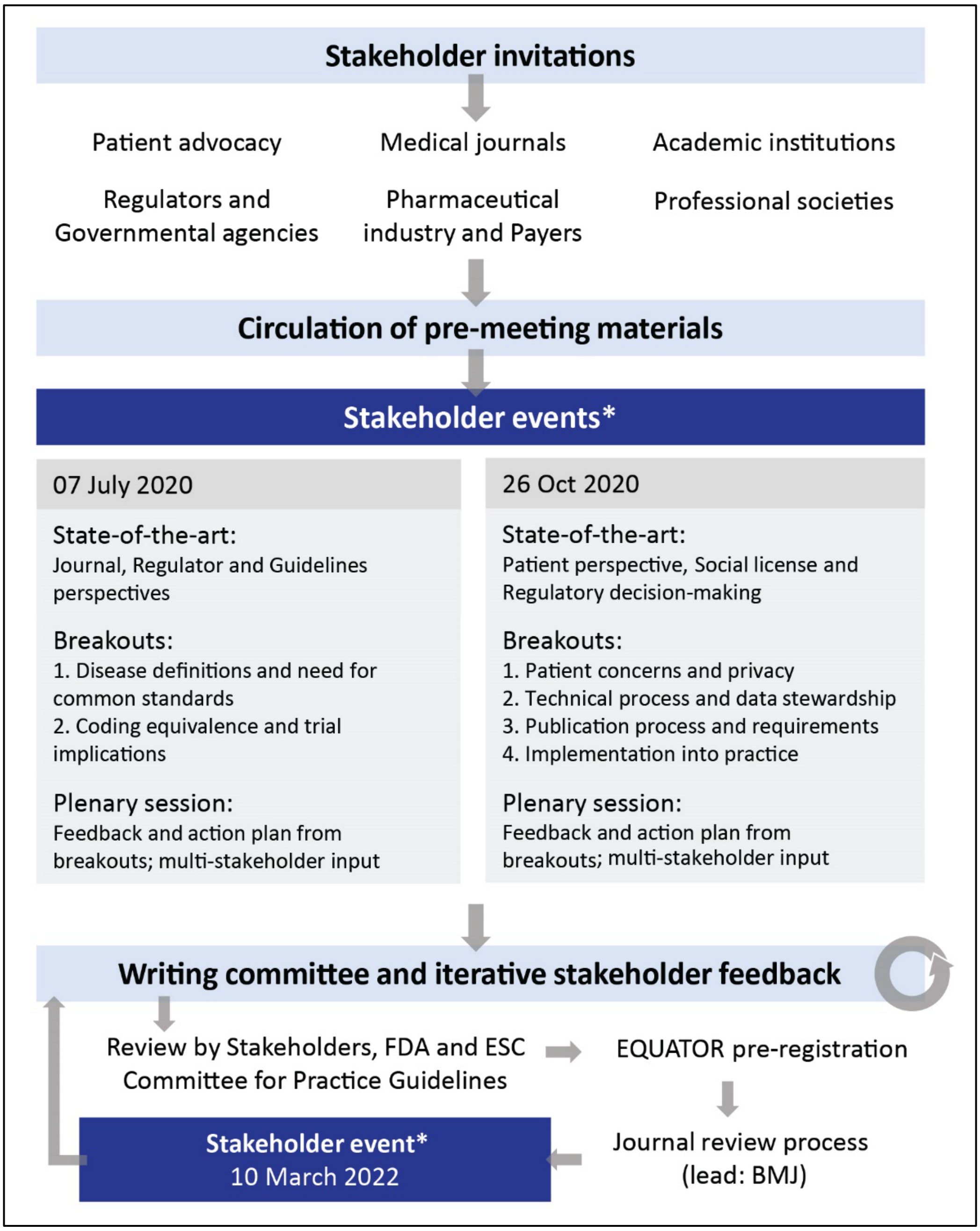

* Stakeholder events were converted to virtual meetings due to the coronavirus pandemic.

B) Design elements for the CODE-EHR framework mapped to EQUATOR items

The following table outlines the design of the CODE-EHR framework, listed according to the EQUATOR network (Enhancing the QUAlity and Transparency Of health Research) approach [1].

<table>
<tr><th colspan="2">EQUATOR item</th><th>CODE-EHR framework</th></tr>
<tr><td rowspan="11">Initial steps</td><td colspan="2">1. Identify the need for a guideline</td></tr>
<tr><td>1.1. Develop new guidance</td><td>A compelling rationale was identified through the BigData@Heart Innovative Medicines Initiate public-private partnership. There is a lack of consistent advice for researchers using electronic healthcare record (EHR) studies, and no minimum standards available to assist in determining the value, transparency and social license of current studies. There is also a clear need to support the wider implementation of good quality real-world data research, based on the FAIR data principles (Findable, Accessible, Interoperable and Reusable).</td></tr>
<tr><td>1.2. Extend existing guidance</td><td>This framework builds on and complements previous reporting checklists [2-5], and extends to provide a focus on structured healthcare data with a common set of minimum standards that can be applied across different specialities to aid in the design and reporting of research. Preferred standards provide a direction of travel for all future structured healthcare data studies.</td></tr>
<tr><td>1.3. Implement existing guidance</td><td>Not applicable – objectives are distinct from previous reporting guidelines.</td></tr>
<tr><td colspan="2">2. Review the literature</td></tr>
<tr><td>2.1. Identify previous relevant guidance</td><td rowspan="3">Launched in March 2017, BigData@Heart includes a broad range of clinical, academic and industry partners across Europe, supported by Patient Advocacy through the European Heart Network, and at the professional level by the European Society of Cardiology. The project brings together 19 leading organisations in the healthcare sector: 5 EFPIA companies; 11 Universities, research organisations, public bodies, non-profit groups; and 3 small or mid-sized companies (https://www.bigdata-heart.eu/Partners).
In 2018-2019 literature reviews were performed and additional investigations were conducted with a broad stakeholder perspective on the topic of structured healthcare data. A range of other systematic reviews and meta-analyses were also performed in areas supporting the use of big data and advanced analytics in healthcare (see www.bigdata-heart.eu/Publications for full list of publications).</td></tr>
<tr><td>2.2. Seek relevant evidence on the quality of reporting in published research articles</td></tr>
<tr><td>2.3. Identify key information related to the potential sources of bias in relevant studies.</td></tr>
<tr><td colspan="2">3. Obtain funding for the guideline initiative</td></tr>
<tr><td></td><td>The BigData@Heart project has received funding from the Innovative Medicines Initiative 2 Joint Undertaking under grant agreement No 116074. This Joint Undertaking receives support from the European Union's Horizon 2020 research and innovation programme, and the European Federation of Pharmaceutical Industries and Associations (EFPIA).
The CODE-EHR framework was funded through the BigData@Heart programme, with stakeholder meetings organised by the European Society of Cardiology, a not-for-profit professional organisation including 57 countries (www.escardio.org).</td></tr>
</table>

Pre-meeting	4. Identify participants	
		Participants were identified in a multi-stage process: Firstly, identification of a small group of attendees from the project consortium and outside the project consortium; from October 2019/December 2019 invitations to key experts identified by this group, covering a range of stakeholders; January/February 2020 invitations to additional experts following generation of objectives and agendas. Invited stakeholders for each meeting consisted of regulators, journal editors, representatives from industry, funders, payers, patient representatives, academics, clinicians and the ESC professional organisation.<br><br>First stakeholder meeting on 7 July 2020: 39 participants (focus on journals, regulators and clinical practice guidelines).<br>Second stakeholder meeting on 26 October 2020: 48 participants (focus on patient perspective, social license and regulatory decision-making).<br>Third stakeholder meeting on 10 March 2022: 23 participants (focus on revisions to the CODE-EHR checklist).
	**5. Conduct a Delphi exercise**	
		The proportion of invited participants who attended at least one meeting was 78%. All stakeholders, including those unable to join meetings, have been involved at all stages, including asking for feedback and comments on reports and meeting summaries, the checklist and this publication. In particular, there were 5 rounds of revision with 97 individual feedback responses.<br>We used a variation of the Delphi method (estimate-talk-estimate) to allow experts to interact between iterations (i.e. not anonymous so that multi-stakeholder input was visible at each stage). Statements and advisories were worked on within breakout groups, amended offline iteratively, and re-presented in plenary sessions with the Stakeholder groups. The checklist did undergo an anonymous survey to capture unbiased feedback, which was later discussed within further breakout sessions to close the Delphi loop.
	**6. Generate a list of items for consideration at the face-to-face meeting**	
		In the last few years, the BigData@Heart consortium's work has focused on advancing data science that underpins the better use of clinical and coded healthcare data, with the aim of improving global cardiovascular health and implementing these approaches into routine practice. After multiple discussions across work packages in the consortium, the need for a roadmap to improve the use of coded EHR data for clinical research was clearly evident. The consortium conducted a systematic review to identify and map real-world data sources for major cardiovascular diseases, highlighting current gaps in transparency and data access (www.karger.com/Article/FullText/520674).[6]<br><br>Items for the stakeholder meetings were firstly discussed during the coordination meetings, management group meetings and consortium meetings of the BigData@Heart project. A joint working group of Work Package 2 (Disease understanding and outcomes definition) and Work Package 4 (Enrichment) was set up, with contribution from all consortium members and support from Work Package 6 (Communications of results and guidance documents). With regards to the checklist, attendees/experts at the first stakeholder meeting were requested to list major elements they advised for inclusion in the eventual framework; later discussed and iteratively developed at the second and third stakeholder meetings.

	**7. Prepare for the meeting**	
	7.1. Decide size and duration of the meeting	A meeting size of 30-50 participants was deemed suitable to gain a broad stakeholder opinion on the development of the CODE-EHR framework.<br>See item 4 for numbers attending each meeting.
	7.2. Develop meeting logistics	Due to the coronavirus pandemic, the face-to-face meeting was converted into a videoconference meeting, but breakouts and plenary sessions were maintained.
	7.3. Develop meeting agenda<br><br>*7.3.1. Consider presentations on relevant background topics, including summary of evidence.*<br><br>*7.3.2. Plan to share results of Delphi exercise, if done.*<br><br>*7.3.3. Invite session chairs.*	Agenda of the first stakeholder meeting:<br>https://www.escardio.org/Research/Big-Data-Heart/bigdata-heart-stakeholder-event-july-2020<br>The agenda included:<br>(1) State-of-the-art presentations by key opinion leaders<br>• Journal Editor perspective [Rupa Sarkar, Lancet Digital Health]<br>• Regulator perspective [Xavier Kurz, EMA]<br>• Professional & Guidelines perspective [Stephan Achenbach, ESC]<br>(2) Two breakout sessions with key questions on the landscape of contemporary real-world data evidence:<br>• Definitions of disease in EHRs & the need for common standards<br>• Coding equivalence and implications for clinical trials<br>(3) Plenary session.<br><br>Agenda of the second stakeholder meeting:<br>https://www.escardio.org/Research/Big-Data-Heart/bigdata-heart-stakeholder-event-october-2020<br>The agenda included:<br>(1) State-of-the-art presentations by key opinion leaders<br>• Patient perspective [Birgit Beger, European Heart Network]<br>• Social licence [Ghislaine van Thiel, UMC Utrecht]<br>• Regulatory decision making [Robert Kazmierski, FDA].<br>(2) Four breakout sessions:<br>• Patient concerns & privacy<br>• Technical process & data stewardship<br>• Publication process & requirements<br>• Implementation into practice of EHR studies (regulatory to practice)<br>(3) Plenary session.<br><br>Agenda of the third stakeholder meeting:<br>The agenda included a summary of iterative feedback on the framework in plenary session, followed by two breakout sessions to assess the revised version of the checklist and discuss feedback from the Delphi process, followed by a plenary wrap-up session [website pending].
	7.4. Prepare materials to be sent to participants prior to meeting	Materials were sent to all delegates prior to meetings and at regular points throughout the development of the CODE-EHR framework.<br>Briefing conferences were organised prior to each meeting, including the Chairs, speakers, moderators and technical/logistic staff.
	7.5. Arrange to record the meeting	Meetings were recorded for internal purposes to generate minutes and reports.

The face-to-face consensus meeting itself	**8. Present and discuss results of pre-meeting activities and relevant evidence**	
	8.1. Discuss the rationale for including items in the checklist.	The rationale was developed during the first stakeholder meeting (July 2020). Based on the July breakout discussions, a 'standards framework' for the use of EHR coded data in studies (to complement existing reporting checklists) was drafted and discussed at the second stakeholder meeting in October 2020. Authors and stakeholders, who contributed to the previous events were invited to the third stakeholder meeting in March 2022, to finalise the CODE-EHR framework for the use of structured healthcare data in clinical research.
	8.2. Discuss the development of a flow diagram.	Drafted by the writing group; presented during the stakeholder meeting on 10 March 2022; subsequent iterative development via email.
	8.3. Discuss strategy for producing documents; identify who will be involved in which activities; discuss authorship.	All stakeholders and experts that contributed to the discussions were invited as co-authors for the paper. All named authors were asked to complete the ICMJE document for transparency in conflicts of interest.<br>All attendees are listed in the Acknowledgements section of the paper.<br>Output from stakeholder events were transcribed by a professional medical writer. The leads for the project (Professors Kotecha and Asselbergs) managed the writing committee.
	8.4. Discuss knowledge translation strategy One of the last major sessions of the meeting should be devoted to issues pertaining to disseminating the reporting guideline.	At the second stakeholder meeting in October 2020, delegates agreed the need and rationale for publication of a new framework to support better quality and transparency for research using structured healthcare data. A 'white paper' was proposed based on the discussions held in the first two stakeholder meetings, to accompany the new checklist. A subsequent teleconference was held with the Editor-in-Chiefs of the journals participating in the stakeholder events, and a strategy formulated for submission which was later ratified by the stakeholder group.<br>The final report was circulated to all attendees of the stakeholder events and went through a period of iteration using a virtual sharepoint to gain comments and edits from all stakeholders simultaneously.
Post-meeting Activities	**9. Develop the guidance statement**	
		The process of developing the checklist required multiple iterations, including via plenary sessions and breakouts in meetings, email correspondence, and comments through the virtual sharepoint.<br>A document to report on the rationale for developing the checklist (this manuscript) was developed from the meeting reports and iterative processes as discussed above.<br>The document underwent independent review by the US Food & Drug Administration and the European Society of Cardiology Committee for Practice Guidelines.<br>The BMJ accepted lead responsibility for peer review. Following the peer review process, changes to the document and checklist were drafted by the writing committee, and then edited and ratified by the stakeholder group following a meeting in March 2022 and email/sharepoint comments. An anonymous survey was conducted, and the checklist content approved through voting.

	9.1. Pilot test the checklist.	Pilot testing was performed using two studies of linked EHR data in the BigData@Heart consortium, a public-private partnership funded by the European Union Innovative Medicines Initiative. The testing led to improvements in the ease of completing the checklist, subsequently approved by the stakeholder group as discussed above. Comments were also received by research fellows working on EHR studies across the BigData@Heart partners, with feedback incorporated into the design of the framework.
	**10. Develop an explanatory document**	
		Advice for completion of the checklist was developed by the writing committee and then circulated to the stakeholder group for iterative development. This is presented in Appendix 2, providing help with best practice and worked examples. As the CODE-EHR framework is designed to act as simple tool for better quality and transparency, the consortium did not believe that a separate and detailed "Explanation and Elaboration" document was necessary; indeed we hope that researchers can readily appreciate the minimal standards listed and quickly report on whether they have met them. Word and PDF versions of the checklist have been provided with form-fields to aid rapid completion. The whole process (including the 'preferred' items) has been designed to act as a driver for better future design of studies that use structured healthcare data.
	**11. Develop a publication strategy**	
	11.1. Consider multiple and simultaneous publications.	The publication strategy was developed in coordination with Editors from the BMJ, The Lancet, Lancet Digital and European Heart Journal (BMJ leading).

Post-publication EQUATOR items will be presented in future updates: 12. Seek and deal with feedback and criticism; 13. Encourage guideline endorsement; 14. Support adherence to the guideline; 15. Evaluate the impact of the reporting guidance; 16. Develop web site; 17. Translate guideline; 18. Update guideline.

References

1. D Moher, KF Schulz, I Simera, DG Altman. Guidance for developers of health research reporting guidelines. *PLoS Med.* 2010;7:e1000217.
2. KF Schulz, DG Altman, D Moher, C Group. CONSORT 2010 statement: updated guidelines for reporting parallel group randomised trials. *BMJ.* 2010;340:c332.
3. E von Elm, DG Altman, M Egger, SJ Pocock, PC Gotzsche, JP Vandenbroucke, S Initiative. The Strengthening the Reporting of Observational Studies in Epidemiology (STROBE) statement: guidelines for reporting observational studies. *Ann Intern Med.* 2007;147:573-7.
4. EI Benchimol, L Smeeth, A Guttmann, et al. The REporting of studies Conducted using Observational Routinely-collected health Data (RECORD) statement. *PLoS Med.* 2015;12:e1001885.
5. AW Chan, JM Tetzlaff, DG Altman, et al. SPIRIT 2013 statement: defining standard protocol items for clinical trials. *Ann Intern Med.* 2013;158:200-7.
6. R Studer, C Sartini, K Suzart-Woischnik, et al. Identification and Mapping Real-World Data Sources for Heart Failure, Acute Coronary Syndrome, and Atrial Fibrillation. *Cardiology.* 2022;147:98-106.
